# A cluster-based analysis evaluating the impact of comorbidities in fibrotic interstitial lung disease

**DOI:** 10.1186/s12931-020-01579-7

**Published:** 2020-12-07

**Authors:** Alyson W. Wong, Tae Yoon Lee, Kerri A. Johannson, Deborah Assayag, Julie Morisset, Charlene D. Fell, Jolene H. Fisher, Shane Shapera, Andrea S. Gershon, Gerard Cox, Andrew J. Halayko, Nathan Hambly, Helene Manganas, Mohsen Sadatsafavi, Pearce G. Wilcox, Teresa To, Veronica Marcoux, Nasreen Khalil, Martin Kolb, Christopher J. Ryerson

**Affiliations:** 1 Department of Medicine, University of British Columbia, Vancouver, BC Canada; 2 Centre for Heart Lung Innovation, St. Paul’s Hospital, Vancouver, BC Canada; 3 Respiratory Evaluation Sciences Program, Collaboration for Outcomes Research and Evaluation, Faculty of Pharmaceutical Sciences, University of British Columbia, Vancouver, BC Canada; 4 Department of Medicine, University of Calgary, Calgary, AB Canada; 5 Department of Medicine, McGill University, Montreal, QC Canada; 6 Département de Médecine, Centre Hospitalier de l’Université de Montréal, Montreal, QC Canada; 7 Department of Medicine, University of Toronto, Toronto, ON Canada; 8 Institute for Clinical Evaluative Sciences, Toronto, ON Canada; 9 Child Health Evaluative Sciences, The Hospital for Sick Children, Toronto, ON Canada; 10 Dalla Lana School of Public Health, University of Toronto, Toronto, ON Canada; 11 Department of Medicine, Firestone Institute for Respiratory Health, The Research Institute of St. Joe’s Hamilton, St. Joseph’s Healthcare, McMaster University, Hamilton, ON Canada; 12 Department of Internal Medicine, University of Manitoba, Winnipeg, MB Canada; 13 Department of Medicine, University of Saskatchewan, Saskatoon, SK Canada

**Keywords:** Interstitial lung disease, Pulmonary fibrosis, Comorbidities, Outcomes

## Abstract

**Background:**

Comorbidities are frequent and have been associated with poor quality of life, increased hospitalizations, and mortality in patients with interstitial lung disease (ILD). However, it is unclear how comorbidities lead to these negative outcomes and whether they could influence ILD disease progression. The goal of this study was to identify clusters of patients based on similar comorbidity profiles and to determine whether these clusters were associated with rate of lung function decline and/or mortality.

**Methods:**

Patients with a major fibrotic ILD (idiopathic pulmonary fibrosis (IPF), fibrotic hypersensitivity pneumonitis, connective tissue disease-associated ILD, and unclassifiable ILD) from the CAnadian REgistry for Pulmonary Fibrosis (CARE-PF) were included. Hierarchical agglomerative clustering of comorbidities, age, sex, and smoking pack-years was conducted for each ILD subtype to identify combinations of these features that frequently occurred together in patients. The association between clusters and change in lung function over time was determined using linear mixed effects modeling, with adjustment for age, sex, and smoking pack-years. Kaplan Meier curves were used to assess differences in survival between the clusters.

**Results:**

Discrete clusters were identified within each fibrotic ILD. In IPF, males with obstructive sleep apnea (OSA) had more rapid decline in FVC %-predicted (− 11.9% per year [95% CI − 15.3, − 8.5]) compared to females without any comorbidities (− 8.1% per year [95% CI − 13.6, − 2.7]; p = 0.03). Females without comorbidities also had significantly longer survival compared to all other IPF clusters. There were no significant differences in rate of lung function decline or survival between clusters in the other fibrotic ILD subtypes.

**Conclusions:**

The combination of male sex and OSA may portend worse outcomes in IPF. Further research is required to elucidate the interplay between sex and comorbidities in ILD, as well as the role of OSA in ILD disease progression.

## Introduction

Interstitial lung disease (ILD) is a collection of diseases that lead to varying degrees of inflammation and fibrosis of the pulmonary parenchyma [1]. Common fibrotic ILDs include idiopathic pulmonary fibrosis (IPF), connective tissue disease-associated ILD (CTD-ILD), fibrotic hypersensitivity pneumonitis (HP), and unclassifiable ILD. Comorbidities are prevalent in patients with ILD and occur at different frequencies among the various ILD subtypes [2]. The most common comorbidities found in patients with fibrotic ILD include gastroesophageal reflux disease, chronic obstructive pulmonary disease, and diabetes [2].

Comorbidities have been associated with reduced quality of life, lower functional capacity, increased hospitalization rates, and mortality in patients with ILD [3–6]. However, it is unclear whether comorbidities lead to negative outcomes because of their direct health effects or if they could also influence ILD progression. This latter possibility is suggested by the variable impact of comorbidities on all-cause mortality across different ILD subtypes [7], but with limited evidence currently available from large prospective cohorts. To address this uncertainty, we sought to identify and characterize clusters of patients with fibrotic ILD based on the presence of similar comorbidities. We further investigated the association between these clusters and the rate of lung function decline and overall mortality.

## Methods

### Study population and overview

The CAnadian REgistry for Pulmonary Fibrosis (CARE-PF) is a prospective cohort of patients with all subtypes of fibrotic ILD who are over age 18, are able to provide informed consent, and can complete questionnaires in English or French [8]. At the time of data export, there were eight participating centers that came from five of the six most populous Canadian provinces. Patients with IPF, CTD-ILD, fibrotic HP, and unclassifiable ILD were included, representing the four most common fibrotic ILD subtypes in CARE-PF [2]. Diagnoses were made at ILD centres with access to multidisciplinary teams comprised of ILD clinicians, chest radiologists, and lung pathologists. There were no exclusion criteria for this sub-study. Ethics approval for this project was obtained at all CARE-PF centres (coordinating centre: University of British Columbia #H19-01989).

### Measurements

Baseline demographic data and smoking history were obtained from patient-completed questionnaires and chart reviews. Patient-completed surveys and clinical records from the date of the first ILD clinic visit were used to record comorbidities as defined by the Charlson Comorbidity Index (CCI), which is associated with mortality in multiple patient populations and diseases [9, 10]. The presence or absence of the 19 major CCI comorbidities were each verified by trained research personnel, rather than using International Classification of Diseases (ICD) codes in order to minimize diagnostic inaccuracies from coding errors. Patients attending the ILD clinic were typically seen every 3–6 months with pulmonary function tests (PFTs) performed at each visit.

### Outcomes

The primary outcome was the annual change in forced vital capacity (FVC) %-predicted, which was calculated based on serial PFTs performed from the time of initial consultation in the ILD clinic using established standards at all sites [11]. Time to death or lung transplantation was the secondary outcome, which was defined from the time of diagnosis.

### Statistical analyses

Within each fibrotic ILD subtype, hierarchical agglomerative clustering [12] was conducted to identify clusters of patients based on the presence of similar comorbidities, age, sex, and smoking pack-years. These variables were standardized to a unit interval ranging from 0 to 1. The hierarchical agglomerative clustering method began with each patient within his or her own cluster. Two clusters were merged if their combination resulted in a new cluster with the lowest distance (intracluster variation) between observations [12]. The distance between clusters was then re-calculated and the next pair of clusters were merged. This process was repeated until a single cluster that contained all patients remained. The final number of clusters for each ILD subtype was not pre-specified, with the optimal number of clusters determined using the average silhouette method [13]. This method measures how well observations are clustered by estimating the average distance (width) between clusters. An average silhouette width is calculated for each cluster option and ranges between -1 and 1, with 1 representing very well clustered observations, 0 meaning observations lie between two clusters, and negative values representing observations that are likely placed in the wrong cluster [14]. A sensitivity analysis was conducted using the gap statistic method to ensure that the optimal number of clusters was similar using different techniques [15]. The gap statistic is the difference between the intracluster variation and the maximum variation between observations when there are no clusters (reference). The gap statistic is calculated for each cluster option. The optimal number of clusters will have a larger gap statistic, which means that the intracluster variation is lower and further away from the reference.

The annual rate of change in FVC was compared between clusters within each ILD subtype using linear mixed effect models. The models were adjusted for age, sex, body mass index (BMI), and smoking pack-years since clustering may not fully address confounding by these covariates. Comparisons between all combinations of clusters were performed. The models included a random intercept and random slope to account for between-patient variability over and beyond the variability induced by included covariates. Kaplan Meier curves were used to assess differences in survival between clusters. All statistical analyses were performed using R (version 3.5.1) [16]. A two-sided p-value < 0.05 was considered statistically significant.

## Results

The study cohort had a total of 1,480 patients (Table 1), including 330 with IPF, 672 with CTD-ILD, 135 with fibrotic HP, and 343 with unclassifiable ILD. Median follow-up was 3.3 years (IQR 1.9–5.5 years), and was similar across ILD subtypes. There were a total of 147 deaths, with 57 in patients who had IPF, 47 in CTD-ILD, 10 in fibrotic HP, and 33 in unclassifiable ILD. These populations had a total of 10,005 PFTs, with 1843, 5288, 963, and 1911 respectively in each ILD subtype. The median number of PFTs per patient was 5 (IQR 3–9). On average, the IPF cohort was older, had a higher proportion of males, and had a greater number of smoking pack-years compared to the other ILD subtypes. Baseline disease severity was mild-to-moderate for all ILD subtypes, with mean FVC ranging from 75 to 81%-predicted and diffusing capacity of the lung for carbon monoxide from 60 to 65%-predicted.

**Table 1:** Baseline patient demographics

Characteristics	IPF (n = 330)	CTD-ILD (n = 672)	Fibrotic HP (n = 135)	Unclassifiable (n = 343)
Age, years	68 ± 8	57 ± 13	62 ± 12	65 ± 12
Male	246 (75%)	209 (31%)	59 (44%)	185 (54%)
BMI, kg/m^2^	29 ± 5	28 ± 6	31 ± 6	30 ± 6
Current or past smoking	263 (80%)	335 (50%)	73 (54%)	217 (63%)
Smoking pack-years	21 (2–37)	0 (0–18)	4 (0–21)	8 (0–28)
Baseline lung function				
FVC, %-predicted	80 ± 18	77 ± 19	75 ± 19	81 ± 20
DLCO, %-predicted	60 ± 18	63 ± 20	62 ± 19	65 ± 21

Values represent mean ± standard deviation, number (percent), or median (interquartile range)

*BMI* body mass index, *FVC* forced vital capacity, *DLCO* diffusing capacity of the lung for carbon monoxide, *IPF* idiopathic pulmonary fibrosis, *CTD-ILD* connective tissue disease-associated ILD, *HP* hypersensitivity pneumonitis

### Frequency of comorbidities

**Fig. 1:**
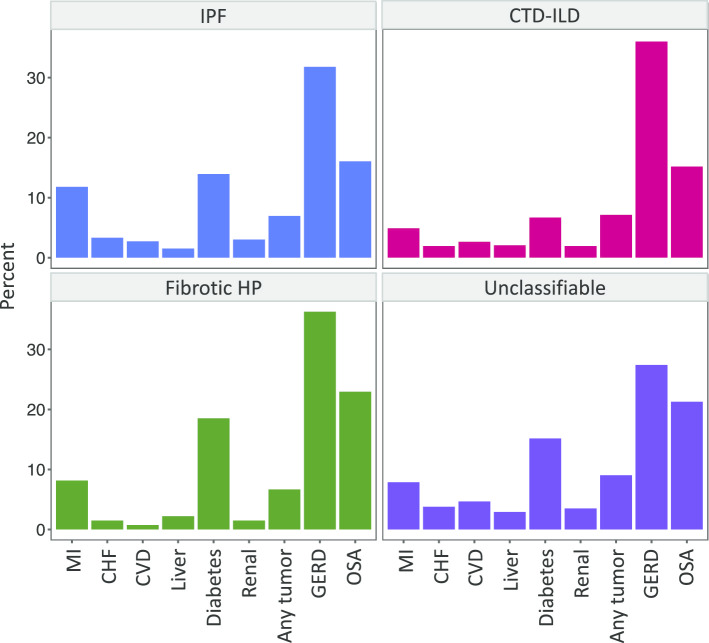
Prevalence of baseline comorbidities among ILD subtypes. Abbreviations: *MI* myocardial infarction; *CHF* congestive heart failure; *CVD* cerebrovascular disease; *GERD* gastroesophageal reflux disease; *OSA* obstructive sleep apnea

The frequency of comorbidities in each ILD subtype is shown in Fig. 1. The most prevalent comorbidities among all ILD subtypes were diabetes, gastroesophageal reflux disease (GERD), and obstructive sleep apnea (OSA). Myocardial infarction occurred in 7% of all patients, with the highest prevalence in IPF (39/330, 12%). A history of malignancy occurred in 7.5% of all patients and was the highest for patients with unclassifiable ILD (31/343, 9%). Congestive heart failure (CHF), cerebrovascular disease, liver disease, and renal disease occurred in < 5% of patients for all ILD subtypes.

### Baseline characteristics of each cluster

**Fig. 2:**
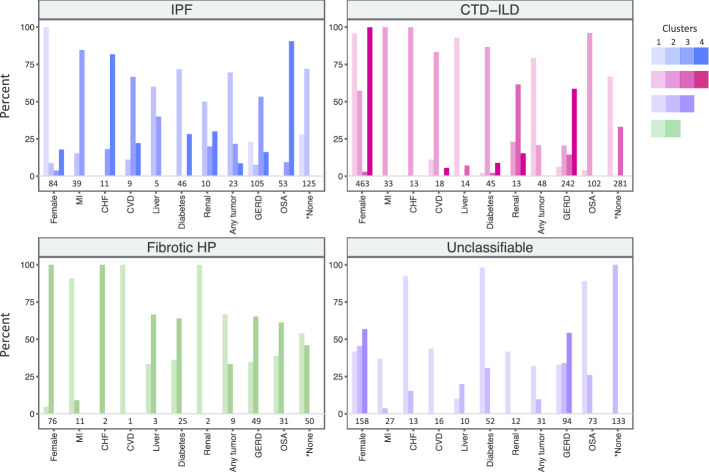
Cluster composition among different ILD subtypes. Clusters are represented by different color bars, with IPF, CTD-ILD, fibrotic HP, and unclassifiable ILD having 4, 4, 2, and 3 clusters each, respectively. The x-axis labels show the total number of patients with a given feature within each ILD subtype. The percentage of females and the percentage of patients with each comorbidity is shown for each cluster on the y-axis. For example, of the 39 patients with IPF who had a MI, 15% were in cluster 2 and 85% were in cluster 3. *Denotes patients who did not have any comorbidities of interest. Abbreviations: *MI* myocardial infarction; *CHF* congestive heart failure; *CVD* cerebrovascular disease; *GERD* gastroesophageal reflux disease; *OSA* obstructive sleep apnea

Using hierarchical agglomerative clustering, the optimal number of clusters for IPF, CTD-ILD, fibrotic HP, and unclassifiable ILD were 4, 4, 2, and 3, respectively (Fig. 2). Sensitivity analysis for the optimal number of clusters was similar using the gap statistic method. Mean age and smoking pack-years were similar among clusters within each ILD subtype (Additional file 1: Table S1), while sex and the presence of specific comorbidities varied and were the main features that distinguished clusters (Table 2). The distribution of major CTD subtypes among the CTD clusters is shown in Additional file 1: Table S2.

**Table 2:** Rate of FVC decline between ILD clusters

ILD subtype	Cluster	Number of patients		Predominant cluster features (sex, comorbidities)	Annual change in FVC (%-predicted)	95% CI
IPF	1	59		Female, none	− 8.1	− 13.6, − 2.7
	2	136		Male, none	− 8.8	− 11.3, − 6.3
	3	79		Male, GERD or MI	− 9.0	− 11.8, − 6.1
	4	56		Male, OSA	− 11.9	− 15.3, − 8.5
CTD-ILD	1	237		Female, any tumor or none	− 0.9	− 2.9, 1.1
	2	157		Female or male, cardiovascular risk factors*	− 1.4	− 3.2, 0.3
	3	136		Male, none	0.4	− 1.2, 1.9
	4	142		Female, GERD	− 0.2	− 1.5, 1.0
Fibrotic HP	1	62		Male, none	− 0.04	− 8.9, 8.8
	2	73		Female, GERD or OSA	1.0	− 5.7, 7.8
Unclassifiable	1	103		Male, Diabetes or OSA	1.1	− 3.3, 5.3
	2	189		Female or male, none	− 1.4	− 3.9, 1.1
	3	51		Female or male, GERD	− 0.2	− 3.1, 2.6

In IPF, cluster 4 had a significantly greater rate of FVC decline compared to cluster 1 (p = 0.03). There were no significant differences between the other clusters

*CI* confidence interval, *FVC* forced vital capacity, *GERD* gastroesophageal reflux disease, *MI* myocardial infarction, *OSA* obstructive sleep apnea

*Cardiovascular risk factors include myocardial infarction, congestive heart failure, cerebrovascular disease, diabetes, and obstructive sleep apnea

### Change in FVC over time across clusters

**Fig. 3:**
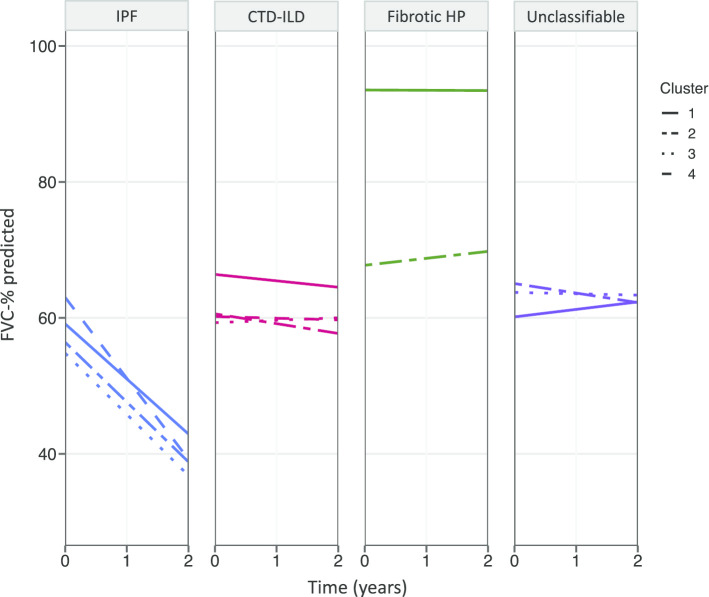
Rate of FVC decline over 2 years from the time of ILD diagnosis by cluster. IPF cluster 4 had significantly greater rate of lung function decline compared to cluster 1 (p = 0.03). There was no statistically significant difference in rate of lung function decline between clusters in other ILD subtypes. Abbreviations: *FVC* forced vital capacity

Baseline FVC was similar among all clusters within each ILD subtype with the exception that patients in HP cluster 1 (predominantly males) had a 26% higher absolute baseline FVC %-predicted compared to HP cluster 2 (predominantly females). Patients with IPF had the greatest rate of FVC decline compared to other ILD subtypes (Fig. 3), after adjusting for prespecified covariates. Within each ILD subtype, the only significant difference across clusters in rate of FVC change was between IPF clusters 1 and 4. The rate of FVC decline in IPF cluster 4 (males with OSA) was 11.9% per year (95%CI 8.5–15.3) compared to IPF cluster 1 (females without comorbidities), which was 8.1% per year (95% CI 2.7–13.6; p = 0.03). The mean FVC decline for IPF clusters 2 and 3 was 8.8% and 9% annually. There were no significant differences in the rate of FVC change across clusters in CTD-ILD, fibrotic HP, or unclassifiable ILD.

### Differences in overall survival across clusters

**Fig. 4:**
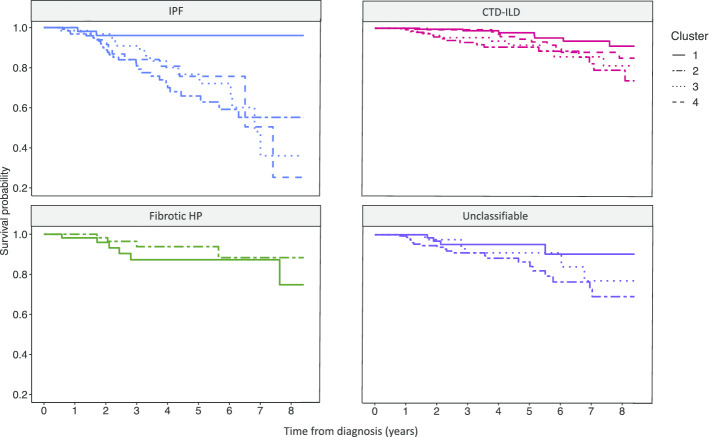
Survival by clusters among ILD subtypes. IPF cluster 1 had significantly higher probability of survival over 8 years compared to the other clusters (p < 0.007). There was no significant difference in survival between clusters in the other ILD subtypes

Patients with IPF had the highest mortality compared to the other ILD subtypes. In IPF, females without comorbidities (cluster 1) had lower mortality compared to males with no comorbidities (cluster 2), GERD or history of MI (cluster 3), and OSA (cluster 4), with over 90% of patients alive at 8 years after diagnosis (p = 0.005 for IPF cluster 1 vs cluster 2, and p = 0.007 for IPF cluster 1 vs clusters 3 or 4). The lowest probability of survival occurred in IPF cluster 4 (males with OSA) with less than 30% of patients still alive at 8 years after diagnosis. There were no differences in survival among the clusters in CTD-ILD, fibrotic HP, and unclassifiable ILD (Fig. 4).

## Discussion

In this study, we used an unsupervised approach to identify clusters based on age, sex, smoking pack-years, and comorbidities in an attempt to identify distinct phenotypes of fibrotic ILD that may be associated with a poor prognosis. The main differences between clusters were patient sex and comorbidities for all ILD subtypes. Among patients who had IPF, we found that a cluster of patients predominantly characterized by males with OSA had a significantly greater rate of FVC decline and worse survival compared to females without comorbidities. There were no significant differences in these outcomes between clusters in other ILD subtypes. These findings suggest the need for further studies exploring potential underlying mechanisms that connect certain comorbidities to ILD progression, and particularly the potential role of OSA.

Cluster analysis is an established approach that has not been widely applied in cohorts of patients with ILD. We used this approach as a novel way to predict outcomes in this population. Cluster analysis offered an unbiased hypothesis-generating approach that allowed the data to identify potentially important relationships between predictor variables, rather than restricting the analysis to preconceived notions held by investigators (e.g., only including interaction terms that investigators believe exist). Through cluster analysis, we were able to explore the complex interplay between several biologic (age, sex, comorbidities) and environmental features (smoking pack-years) that can be used to inform future studies.

Males with OSA had the greatest rate of lung function decline and the worst survival in IPF after adjusting for age, sex, BMI, and smoking pack-years. This finding is similar to a previous study of 92 patients with ILD that showed 65% had at least mild OSA diagnosed by nocturnal polysomnogram, with the presence of OSA similarly being associated with worse progression-free survival [17]. It is unclear whether OSA is a risk factor for IPF or vice versa and the reason for these shared findings requires further investigation. One possibility is that OSA results in hypoxia that disrupts cell and tissue homeostasis, thus resulting in more rapid progression of IPF. A second possibility is that large swings in pleural pressure resulting from obstructive events could stretch alveolar walls and lead to repetitive alveolar epithelial cell injury [18]. These repeated injuries, along with aberrant remodeling of the extracellular matrix, could then lead to the development and progression of interstitial lung disease [19]. Additional studies are needed to confirm and further explore the potential link and physiologic impact between OSA and adverse outcomes in IPF. There have been conflicting data on the impact BMI has on survival in ILD. While some studies have shown an increased risk of acute ILD exacerbations with higher BMI [20], others have shown no association of BMI with survival or even an improved survival in patients with a higher baseline BMI [21, 22]. Therefore, although obesity is a risk factor for OSA [23], it is unclear whether an increased BMI impacts disease progression and survival in patients with ILD.

The other main finding from our study is the minimal difference in outcomes between clusters within each ILD subtype despite previous studies suggesting an association between various comorbidities and reduced survival. Arteriosclerosis, other cardiovascular diseases (e.g., valvular heart disease), malignancy, and GERD have all been associated with reduced survival in IPF [24]. For patients with ILD, the presence of additional comorbidities including renal failure, hypothyroidism, and connective-tissue disorders were also associated with reduced survival [7]. However, our results only identified the associations between males with GERD, history of MI, and OSA with reduced survival in IPF. This may be due to differences in comorbidity prevalence between study cohorts. For example, we did not identify a cluster characterized by diabetes in IPF. However, the prevalence of diabetes in our cohort (14%) was lower compared to other cohorts (33%) [7]. In addition, different methods of diagnosing comorbidities (e.g., diagnosis by clinical evaluation versus ICD codes) and the use of diagnostic thresholds to classify disease in previous studies may contribute to these heterogeneous study findings (e.g., hemoglobin A1c > 6% required to diagnose diabetes). This heterogeneity emphasizes the need for ILD registries to prospectively collect comorbidity data and ideally use the same method of classification in order for studies to be comparable.

This study has several limitations. Although we applied a unique statistical approach to a large prospective cohort, the main findings are exploratory and need to be further studied and externally validated. The ability to analyze big data in growing ILD registries around the world is an important need. We believe cluster analysis will play an important role in ILD research as it allows patterns and relationships to be identified in large data sets, which may not have otherwise been apparent. Our findings support the hypothesis that hypoxia and/or exaggerated swings in intrathoracic pressure related to OSA may be an underlying mechanism for disease initiation, progression, and ultimately death in some patients with ILD. This highlights the need for randomized controlled trials to test potential treatments of OSA in patients with IPF. Our analysis was also limited to the comorbidities available in the Charlson comorbidity index and CARE-PF patient surveys, with the possibility that a more comprehensive and robust assessment for specific comorbidities may have yielded additional findings. In addition, an older patient cohort may have increased comorbidities which affect outcomes. However, if there was a causal link between comorbidities and disease progression, we would have expected our study to show further associations. Regardless, this analysis should be conducted in other patient populations, including those who are older and may have more severe comorbidities. Finally, we were unable to account for varying treatment approaches across different ILD subtypes and patient populations, as well as whether comorbidities were treated or not, given the challenges in identifying on- and off-treatment periods in a diverse real-world population.

## Conclusion

In this large prospective multicenter cohort, we show that GERD, diabetes, and OSA are the most prevalent comorbidities across the major fibrotic ILD subtypes. In IPF, males with OSA had significantly greater lung function decline and worse survival compared to females without comorbidities. Although they should be validated, these findings identify important research questions including whether comorbidities influence underlying pro-fibrotic pathways and whether outcomes improve when comorbidities are treated. We hope that the novel approach used in this analysis will support these future studies in an attempt to better understand causes of disease progression in fibrotic ILD and to develop new therapeutic strategies for these patients.

## Supplementary information

**Additional file 1: Table S1.** Cluster composition for each ILD subtype. **Table S2.** Major types of connective tissue diseases in each cluster.

## Data Availability

The data that support the findings of this study are available on request from the corresponding author. The data are not publicly available due to them containing information that could compromise research participant privacy/consent.

## Abbreviations

BMI: Body mass index
CARE-PF: Canadian Registry for Pulmonary Fibrosis
CCI: Charlson Comorbidity Index
CHF: Congestive heart failure
CTD-ILD: Connective tissue disease-associated ILD
FVC: Forced vital capacity
GERD: Gastroesophageal reflux disease
HP: Hypersensitivity pneumonitis
ILD: Interstitial lung disease
ICD: International Classification of Diseases
IPF: Idiopathic pulmonary fibrosis
MI: Myocardial infarction
OSA: Obstructive sleep apnea
PFT: Pulmonary function test

## References

[1] American Thoracic S, European Respiratory S. American Thoracic Society/European Respiratory Society International Multidisciplinary Consensus Classification of the Idiopathic Interstitial Pneumonias. This joint statement of the American Thoracic Society (ATS), and the European Respiratory Society (ERS) was adopted by the ATS board of directors, June 2001 and by the ERS Executive Committee, June 2001. Am J Respir Crit Care Med. 2002;165(2):277–304. 10.1164/ajrccm.165.2.ats01 11790668

[2] Fisher JH, Kolb M, Algamdi M, Morisset J, Johannson KA, Shapera S (2019). Baseline characteristics and comorbidities in the CAnadian REgistry for Pulmonary Fibrosis. BMC Pulm Med.

[3] Butler SJ, Li LSK, Ellerton L, Gershon AS, Goldstein RS, Brooks D (2019). Prevalence of comorbidities and impact on pulmonary rehabilitation outcomes. ERJ Open Res.

[4] Szentes BL, Kreuter M, Bahmer T, Birring SS, Claussen M, Waelscher J (2018). Quality of life assessment in interstitial lung diseases:a comparison of the disease-specific K-BILD with the generic EQ-5D-5L. Respir Res.

[5] Chen W, FitzGerald JM, Sin DD, Sadatsafavi M, Canadian Respiratory Research N. Excess economic burden of comorbidities in COPD: a 15-year population-based study. Eur Respir J. 2017;50(1):1700393. 10.1183/13993003.00393-2017 28751416

[6] McDonnell MJ, Aliberti S, Goeminne PC, Restrepo MI, Finch S, Pesci A (2016). Comorbidities and the risk of mortality in patients with bronchiectasis: an international multicentre cohort study. Lancet Respir Med.

[7] Schwarzkopf L, Witt S, Waelscher J, Polke M, Kreuter M (2018). Associations between comorbidities, their treatment and survival in patients with interstitial lung diseases—a claims data analysis. Respir Res.

[8] Ryerson CJ, Tan B, Fell CD, Manganas H, Shapera S, Mittoo S (2016). The Canadian Registry for Pulmonary Fibrosis: Design and Rationale of a National Pulmonary Fibrosis Registry. Can Respir J.

[9] Charlson M, Szatrowski TP, Peterson J, Gold J (1994). Validation of a combined comorbidity index. J Clin Epidemiol.

[10] Charlson ME, Pompei P, Ales KL, MacKenzie CR (1987). A new method of classifying prognostic comorbidity in longitudinal studies: development and validation. J Chronic Dis.

[11] Graham BL, Steenbruggen I, Miller MR, Barjaktarevic IZ, Cooper BG, Hall GL, et al. Standardization of spirometry 2019 update. An official American Thoracic Society and European Respiratory Society technical statement. Am J Respir Crit Care Med. 2019;200(8):e70–e88. 10.1164/rccm.201908-1590ST PMC6794117 31613151

[12] Ward JH (1963). Hierarchical grouping to optimize an objective function. J Am Stat Assoc.

[13] Rousseeuw PJ (1987). Silhouettes: a graphical aid to the interpretation and validation of cluster analysis. Comput Appl Math.

[14] Data Novia. Cluster validation statistics: must know methods. Available from: https://www.datanovia.com/en/lessons/cluster-validation-statistics-must-know-methods/. Accessed 13 Sept 2020.

[15] Tibshirani R, Walther G, Hastie T (2000). Estimating the number of clusters in a data set via the gap statistic. J R Statist Soc B.

[16] Ley B, Swigris J, Day BM, Stauffer JL, Raimundo K, Chou W (2017). Pirfenidone reduces respiratory-related hospitalizations in idiopathic pulmonary fibrosis. Am J Respir Crit Care Med.

[17] Troy LK, Young IH, Lau EMT, Wong KKH, Yee BJ, Torzillo PJ (2019). Nocturnal hypoxaemia is associated with adverse outcomes in interstitial lung disease. Respirology.

[18] Tschumperlin DJ, Oswari J, Margulies AS. Deformation-induced injury of alveolar epithelial cells. Effect of frequency, duration, and amplitude. Am J Respir Crit Care Med. 2000;162(2 Pt 1):357–62. 10.1164/ajrccm.162.2.9807003 10934053

[19] King TE, Pardo A, Selman M (2011). Idiopathic pulmonary fibrosis. Lancet.

[20] Kondoh Y, Taniguchi H, Katsuta T, Kataoka K, Kimura T, Nishiyama O (2010). Risk factors of acute exacerbation of idiopathic pulmonary fibrosis. Sarcoidosis Vasc Diffuse Lung Dis.

[21] Pugashetti J, Graham J, Boctor N, Mendez C, Foster E, Juarez M (2018). Weight loss as a predictor of mortality in patients with interstitial lung disease. Eur Respir J.

[22] Alakhras M, Decker PA, Nadrous HF, Collazo-Clavell M, Ryu JH (2007). Body mass index and mortality in patients with idiopathic pulmonary fibrosis. Chest.

[23] Kapur VK, Auckley DH, Chowdhuri S, Kuhlmann DC, Mehra R, Ramar K (2017). Clinical practice guideline for diagnostic testing for adult obstructive sleep apnea: an American Academy of Sleep Medicine Clinical Practice Guideline. J Clin Sleep Med.

[24] Kreuter M, Ehlers-Tenenbaum S, Palmowski K, Bruhwyler J, Oltmanns U, Muley T (2016). Impact of comorbidities on mortality in patients with idiopathic pulmonary fibrosis. PLoS ONE.

