# Supplementary material for: A cluster-based analysis evaluating the impact of comorbidities in fibrotic interstitial lung disease
Source: Respir Res. 2020 Dec 7;21:322. doi: 10.1186/s12931-020-01579-7 (PMC7720501; doi:10.1186/s12931-020-01579-7)
Supplement: Supplementary file 1 — Additional file 1: Table S1. Cluster composition for each ILD subtype. Table S2. Major types of connective tissue diseases in each cluster. [file 12931_2020_1579_MOESM1_ESM.docx]

**Additional**

**Table S1. Cluster composition for each ILD subtype.** Cluster analysis was performed based on patient sex, age, smoking pack-years, and comorbidities. The difference between clusters within each ILD subtype was mainly due to comorbidities and sex, while age and smoking pack years were fairly similar. *cardiovascular risk factors include myocardial infarction, congestive heart failure, cerebrovascular disease, diabetes, and obstructive sleep apnea. Abbreviations: BMI, body mass index; GERD, gastroesophageal reflux disease; MI, myocardial infarction; OSA, obstructive sleep apnea.

| **ILD subtype** | **Cluster** | | **Number of patients** | **Males (%)** | **Mean age (SD)** | **BMI, kg/m^2^**  **(SD)** | **Median smoking pack-years (IQR)** | **Predominant comorbidities** |
| --- | --- | --- | --- | --- | --- | --- | --- | --- |
| **IPF** | 1 | 59 | | 0 | 66 (8.9) | 28.5 (4.8) | 21 (4-35) | None |
|  | 2 | 136 | | 50 | 69 (8.5) | 28.7 (5.0) | 20 (1-33) | None |
|  | 3 | 79 | | 31 | 69 (7.4) | 28.8 (3.7) | 22 (4-41) | GERD or MI |
|  | 4 | 56 | | 19 | 69 (7.3) | 32.1 (5.8) | 21 (0-43) | OSA |
| **CTD-ILD** | 1 | 237 | | 4 | 55 (12.9) | 27.7 (6.5) | 0 (0-13) | Any tumor or none |
|  | 2 | 157 | | 43 | 60 (11.1) | 29.9 (6.0) | 7 (0-25) | Cardiovascular risk factors* |
|  | 3 | 136 | | 97 | 57 (12.1) | 26.9 (4.7) | 0 (0-19) | None |
|  | 4 | 142 | | 0 | 55 (13.5) | 26.9 (5.6) | 0 (0-13) | GERD |
| **Fibrotic HP** | 1 | 62 | | 100 | 62 (11.9) | 29.7 (4.6) | 6 (0-20) | None |
|  | 2 | 73 | | 0 | 61 (12.4) | 31.3 (6.3) | 0 (0-22) | GERD or OSA |
| **Unclassifiable** | 1 | 103 | | 32 | 64 (9.8) | 32.9 (7.2) | 11 (0-31) | Diabetes or OSA |
|  | 2 | 189 | | 56 | 64 (12.8) | 28.8 (5.6) | 7 (0-24) | None |
|  | 3 | 51 | | 12 | 67 (9.9) | 28.1 (3.7) | 3 (0-29) | GERD |

**Table S2.** Major types of connective tissue diseases in each cluster. Data shown are the number of patients with each CTD and corresponding percentages based on the total number of patients with a given CTD are in parentheses. Other CTD refers to undifferentiated CTD. Abbreviations: CTD, connective tissue disease; ILD, interstitial lung disease; SLE, Systemic lupus erythematosus.

| **CTD-ILD Cluster** | **Rheumatoid arthritis** | Sjögren's Syndrome | **Systemic Sclerosis** | **Mixed CTD** | **SLE** | **Myositis** | **Other** |
| --- | --- | --- | --- | --- | --- | --- | --- |
| 1 | 44 (33) | 16 (37) | 55 (28) | 22 (40) | 4 (25) | 44 (36) | 52 (48) |
| 2 | 40 (30) | 12 (28) | 29 (16) | 10 (18) | 6 (38) | 32 (26) | 28 (26) |
| 3 | 38 (28) | 9 (21) | 35 (18) | 5 (9) | 2 (12) | 30 (25) | 17 (15) |
| 4 | 13 (9) | 6 (14) | 74 (38) | 18 (33) | 4 (25) | 15 (13) | 12 (11) |
